# Pelvic Fixation With a Quad-Rod Technique Using S2 Alar Iliac and Medialized Entry Iliac Screws for Long Fusion Constructs

**DOI:** 10.5435/JAAOSGlobal-D-22-00251

**Published:** 2023-08-16

**Authors:** Sherif Sherif, Jeremiah Ling, Ivan Zapolsky, David P. Falk, Kevin Bondar, Vincent Arlet, Comron Saifi

**Affiliations:** From the Department of Orthopaedic Surgery, University of Pennsylvania, Philadelphia, PA (Dr. Sherif, Dr. Zapolsky, Dr. Falk, and Dr. Arlet); the Texas A&M College of Medicine, Bryan, TX (Mr. Ling); Department of Orthopedics and Sports Medicine Houston Methodist Hospital, Houston, TX (Dr. Bondar andDr. Saifi).

**Keywords:** Quad Rod, Medialized Entry Iliac Screw, Adult Spinal Deformity, Sagittal imbalance, Spino-pelvic Fixation

## Abstract

**Purpose:**

Patients with adult spinal deformity (ASD) may have risk factors for nonunion and subsequent instrumentation failure. This study reviews a novel surgical technique for a quad-rod construct to the pelvis using both S2 alar iliac (S2AI) screw fixation and medialized entry iliac screw fixation as described through three separate cases and a review of the literature.

**Methods:**

This technique facilitates alignment of the construct and rod insertion into the tulip heads. The medialized iliac screw technique also avoids the potential soft-tissue complications of the conventional iliac screw bolt given that it is deeper and has more soft-tissue coverage.

**Results:**

Three cases performed by the most senior author (V.A.) in which this novel technique was used are presented in this report along with clinical and radiographic images to educate the reader on appropriate execution of this technique. A review of the existing literature regarding pelvic fixation techniques for ASD was also done.

**Conclusion:**

Quad-rod augmentation of long thoracolumbar spinal constructs with two independent SI anchoring points is potentially an effective technique to increase lumbar sacral construct rigidity, thereby promoting fusion rates and decreasing revision rates. The described technique provides spine surgeons with an additional tool in their armamentarium to treat patients with complex ASD.

## Background

With an increasing mean age of the general population, there has been a growing incidence of symptomatic adult spinal deformity (ASD) necessitating long fusion constructs.^1^ There is increasing evidence of clinical benefit for surgical treatment of ASD^2-4^ However, patients with ASD may have risk factors for nonunion and subsequent instrumentation failure, including the need for three-column osteotomies or a contraindication for biologics such as bone morphogenic protein-2. In addition, given the increasing amount of healthcare expenditure on ASD, minimizing rates of revision surgery would improve cost-effectiveness, which has become an increasing concern among insurers and policy makers.^5^

In 2006, the senior author published a novel method on using a four-rod technique for thoracolumbar reconstruction based on alternating between the Roy Camille screw trajectory (straight in) and Magerl screw trajectory (converging lateral to medial) at each level in the lumbar spine.^6,7^ This was followed by insertion of two iliac screws bilaterally and four rods.^8^ In most cases, this technique required the insertion of an iliac screw on the inner iliac cortex just outside the sacroiliac (SI) joint (modified/medialized anatomic iliac screw) and another screw on the top of the iliac crest (a conventional iliac bolt). One disadvantage of the conventional iliac bolt technique includes prominence, which may lead to wound healing issues, pain, and discomfort, particularly for thin patients.

In addition, the traditional iliac bolt is typically not in line with the cephalad pedicle screw construct which requires an offset connection, and the introduction of yet another interface decreases the strength of the inferior portion of the construct, which is essential for stability.^9,10^

With the advent of the S2 alar iliac (S2AI) screw technique,^11,12,13,14,15,16^ the senior author combined the medialized entry iliac (MEI) screw and the S2AI screw in patients who required pelvic fixation with four screws.

Considering that patients with ASD have risk factors for nonunion and subsequent instrumentation failure, this study aims to describe a novel surgical technique that can be used as a tool to increase lumbar sacral construct rigidity, with the objective of promoting fusion rates in the treatment of patients with complex ASD.

## Methods

### Search Strategy

Two authors conducted separate searches using the following medical databases on January 1, 2022: PubMed (1966-present), SCOPUS (1966-present), and Ovid MEDLINE (1946-present). A focused search strategy was done. Keywords including ‘pelvic fixation,’ ‘S2 alar,’ ‘medialized,’ and ‘long fusion’ were combined with Boolean operators and queried. The results were reviewed in order of relevance. A review of the included references of relevant studies was also done.

### Rational of Treatment

The surgical technique for a quad-rod construct to the pelvis using both S2AI screw fixation and MEI screw fixation is outlined below. This technique facilitates alignment of the construct and rod insertion into the tulip heads. The medialized iliac screw technique also avoids the potential soft-tissue complications of the conventional iliac screw bolt given that it is deeper and has more soft-tissue coverage. The technique allows versatility in selecting the location and direction of bone anchors into the pelvis.^8^ Nonunion and associated rod fracture is a concern with long spinal deformity constructs. The present technique provides additional axial and torsional stability to minimize the risk of rod fracture, particularly in cases of three-column osteotomies.

**Figure 1:**
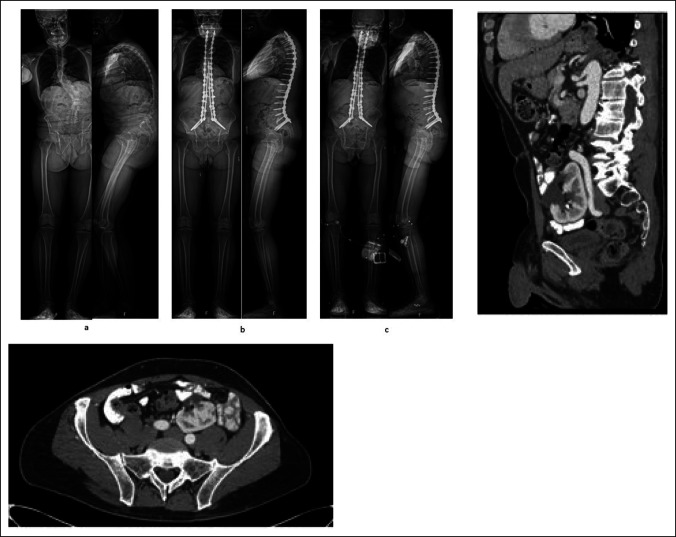
Top Left: AP and lateral full-length standing radiographs (**A**) preoperatively, (**B**) 1 month postoperatively, and (**C**) 20 months postoperatively. Top Right: Preoperative sagittal CT scan demonstrating pelvic kidney and vascular anatomy precluding anterior retroperitoneal approach to the lumbar spine. Bottom: Preoperative axial CT scan demonstrating pelvic kidney and vascular anatomy precluding the anterior retroperitoneal approach to the lumbar spine.

**Figure 2:**
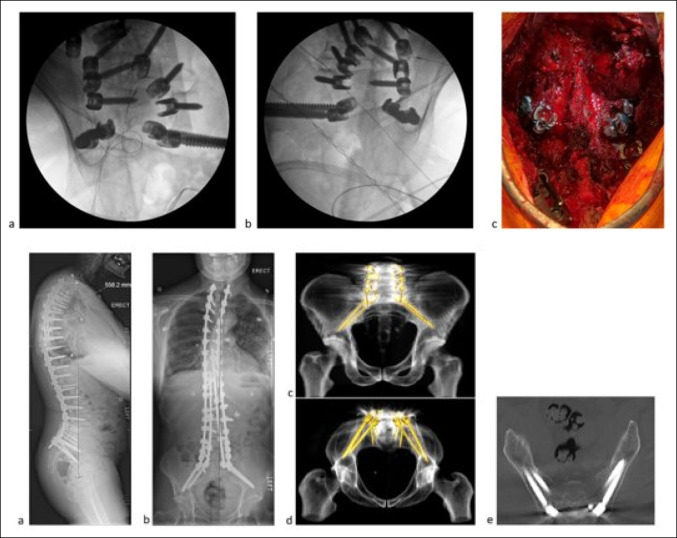
Top: Images showing intraoperative fluoroscopy views of the anatomic iliac screws and S2 pelvic screws, (**A**) left view, (**B**) right view, and (**C**) intraoperative photograph. Bottom: (**A** and **B**) Postoperative standing radiographs and CT scans showing quad-rod technique for sagittal and coronal correction. **C** and **D,** 3D reconstruction from CT scans demonstrating the placement of pelvic instrumentations. **E,** Axial CT scan demonstrating the trajectory of the iliac screws within the pelvic wings.

**Figure 3:**
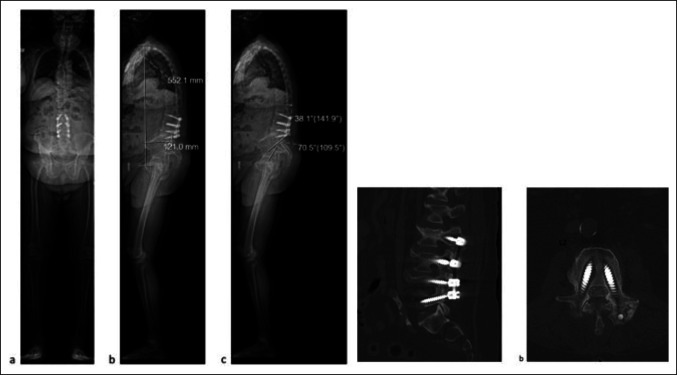
Left: (**A**) Preoperative AP and (**B**) preoperative lateral EOS standing radiographs demonstrating sagittal imbalance with a positive sagittal vertical axis of 12.1 cm. **C,** Preoperative radiograph showing pelvic incidence measured at approximately 70° with a lumbar spine lordosis of approximately 38°. Right: (**A**) Sagittal and (**B**) axial lumbar CT scan demonstrating pseudarthrosis with large halos around the previously placed pedicle screws.

**Figure 4:**
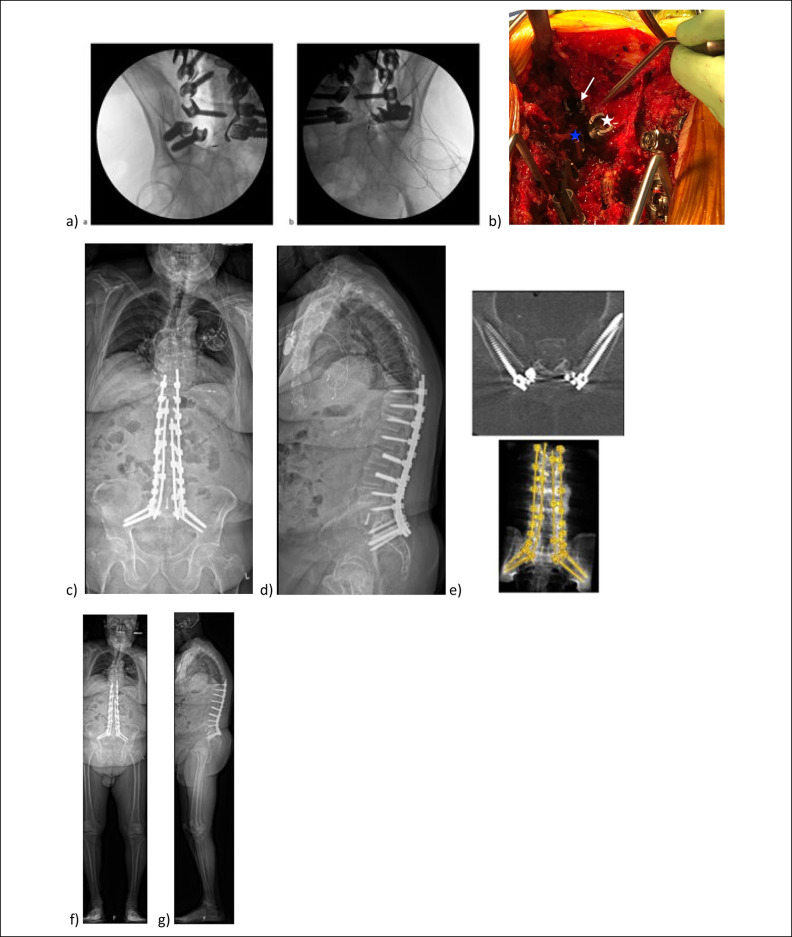
**A,** Bilateral intraoperative teardrop view showing excellent placement of the iliac and S2AI screws on each side, left and right (top is cephalad). **B,** Intraoperative photograph showing right iliac screw (white arrow), right S2AI screw (white star), and right S1 screw (blue star). **C** and **D,** AP and lateral standing radiograph showing restoration of the sagittal balance. **E,** Top: Axial CT cut demonstrating the iliac screw trajectories within the iliac wings; Bottom: 3D reconstruction from CT demonstrating the placement of the pelvic instrumentation. **F,** 6-month postoperative AP radiograph demonstrating maintenance of sagittal balance. **G,** 6-month postoperative lateral radiograph demonstrating maintenance of sagittal balance.

**Figure 5:**
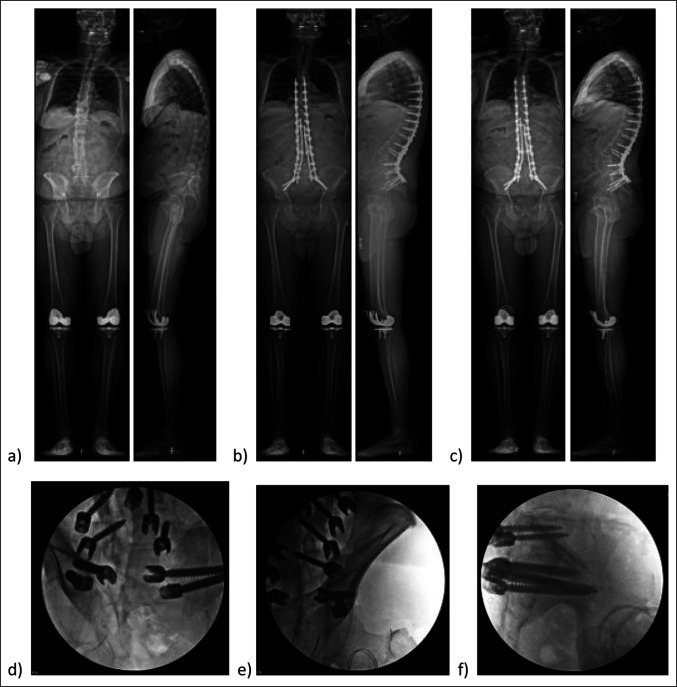
Top: AP and lateral full-length standing radiographs (**A**) preoperatively, (**B**) 1 month postoperatively, and (**C**) 1 year postoperatively. Bottom: Radiographs showing (**D**) left and (**E**) right intraoperative teardrop views and (**F**) lateral view showing placement of the iliac and S2AI screws on each side.

**Figure 6:**
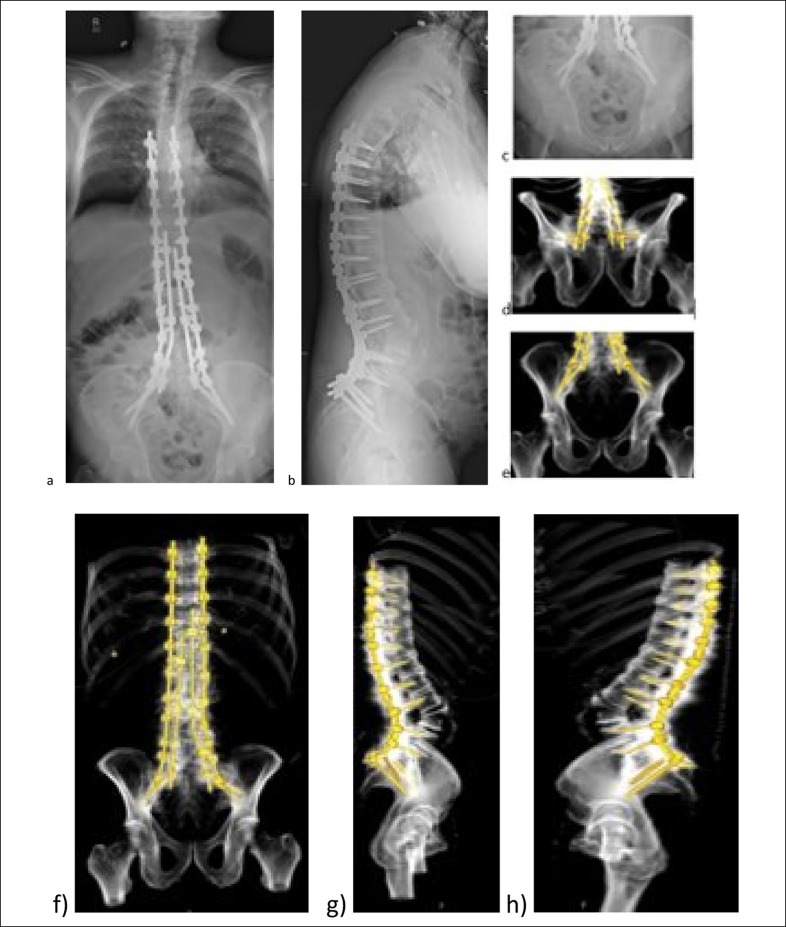
**A,** AP and (**B**) lateral standing radiograph showing restoration of the sagittal balance. **C,** Pelvic inlet view demonstrating the iliac screw trajectories within the iliac wings. **D,** Outlet and (**E**) AP 3D reconstruction from CT demonstrating the placement of the pelvic instrumentation. **F,** PA, (**G**) right, and (**H**) left lateral 3D reconstruction from CT demonstrating the placement of the quad-rod spinal instrumentation.

Three cases in which this novel technique was used are presented in this report (Table 1, Figures 1–6). This project is classified as a case report by the university IRB guidelines and is exempt from IRB review. A detailed augmentation of long spinal constructs with additional rods and pelvic fixation is described. The aim of the technique is to reduce the rates of instrumentation failure and therefore revision surgery.

**Table 1:** Case and Imaging Descriptions

Case #	Description	Imaging
1	(Figures 1 and 2) This 57-year-old male with a history of Parkinson disease (PD) presented with progressive back pain, stiffness, and postural decline over several years. He stated that these symptoms worsened since a motor vehicle collision that occurred 4 years before presentation. He was not able to stand upright, and his postural decline caused him notable discomfort. Previously, he had been treated with physical therapy and anti-inflammatory medications which gave him short-term relief. Parkinson disease symptomatology was well-controlled with antiparkinsonian medications.	Standing global AP and lateral radiograph using EOS imaging (Figure 1) revealed thoracolumbar levoscoliosis with a Cobb angle of 43°. Surgical correction of the deformity was discussed with our patient, and he was to undergo an anterior lumbar interbody fusion with insertion of hyperlordotic cages to provide anterior column support, in addition to assist in sagittal plane correction. This would be supplemented with posterior osteotomies and fixation from T3 to pelvis. Preoperative planning and workup demonstrated the presence of a pelvic kidney and low bifurcation of the retroperitoneal vasculature (Figure 1), precluding the anterior approach. The patient thus underwent a T3-pelvis posterior fusion with posterior column osteotomies and the quad-rod technique for added stability of the construct (Figure 2).
2	(Figures 3 and 4) A 76-year-old man who underwent gastric bypass for morbid obesity, now presents with progressive, worsening, and aching back pain. Despite bariatric surgery, his BMI was 31.5 at presentation. He also had previously undergone L3-5 laminectomy with posterolateral and interbody fusion approximately four years prior for neurogenic claudication and lumbar stenosis. During his postoperative recovery period unfortunately, the patient developed a right-sided foot drop. He underwent revision laminectomy and fusion from L2-S1 approximately 1 year after his index surgery to address his new symptomatology. He improved slightly from his second operation; however, his back pain gradually worsened over time. The pain was deep seated, dull in character, and most severe when standing or ambulating. He had undergone a full spectrum of conservative management including physical therapy and analgesics with limited and temporary relief.	Standing global AP and lateral radiograph using EOS imaging showed sagittal imbalance with a positive sagittal vertical axis of 12.1 cm (Figure 3). Pelvic incidence was measured at approximately 70° with a lumbar spine lordosis of approximately 38°. CT of the lumbar spine also revealed pseudarthrosis at L2-3 and peri-instrumentation halos at L2 and L5 (Figure 3). The patient underwent an L5-S1 anterior lumbar interbody fusion (ALIF) with the use of a 30° hyperlordotic cage for restoration of lumbar lordosis, followed by revision of his posterolateral fusion from T10-pelvis (Figure 4).
3	(Figures 5 and 6) A 71-year-old man with ankylosing spondylitis presents with neck and back pain, worsening forward-leaning posture, and difficulty with horizontal gaze. Patient reported of pain in his low back, bilateral buttock, groin and anterior and posterior thighs. He did not experience numbness but recently began experiencing distal lower extremity paresthesia.	Standing global AP and lateral radiograph using EOS imaging showed sagittal imbalance with a positive sagittal vertical axis of 11.6 cm (Figure 5). Pelvic incidence was measured at approximately 40° with a lumbar spine kyphosis of approximately 6°. Because of the rigidity of the spine secondary to ankylosing spondylitis, a decision was made to use pedicle subtraction osteotomy at L4 to achieve the desired lordosis. The proposed quad-rod technique was used to secure the achieved correction (Figure 5, Figure 6).

## Results

Three cases from our practice demonstrating the described quad-rod technique being used in the care of patients are described (Table 1, Figures 1–6). All cases were done by the senior author.

### Surgical Technique

Anatomic bony landmarks to be exposed:

•Pars interarticularis at each level
•Facet joints at each level
•Transverse processes at each level
•Upper 1/3 of the sacrum including the S1 and S2 sacral foramina
•Sacroiliac joints bilaterally
•Posterior superior iliac spine bilaterally

Dissection is done in a standard subperiosteal fashion until the transverse processes and the upper 1/3 of the sacrum including the S1 and S2 dorsal foramina are exposed. The sacroiliac joints, posterior superior iliac spines, and the iliac crest adjacent to the S1 foramen should also be exposed. A Hohmann retractor can be positioned over the iliac crest to provide excellent exposure of the pelvic instrumentation starting points. To ensure patient safety during this technique, it is not advisable to begin instrumentation until all bony landmarks are clearly visualized given that this technique relies heavily on patient anatomy rather than imaging guidance to place instrumentation. Generally, the only form of imaging necessary to place instrumentation in this technique is preoperative AP and lateral radiographs and a preoperative CT scan (axial cuts). Preoperative CT is recommended because it assists the surgeon in using the anatomic technique to appropriately plan pedicle screw start point, trajectory, and size. CT scan also helps to plan appropriate start point, trajectory, and size of the S2AI and MEI screws because there is some variation in individual patient pelvic anatomy as well as between sexes which affects screw start point and trajectory during insertion. After all bony landmarks are exposed, pedicle instrumentation is begun at L4, followed by L5 and S1 in a caudal progression. Special attention should be paid to the insertion point of the L5 screw, ensuring that it falls on a line between L4 and the S1 pedicle screws. Pelvic instrumentation follows pedicle screw insertion.

### S1 Pedicle Screw Insertion

The S1 sacral screws are placed using the standard bony landmarks. A point midway between the medial aspect of the sacral ala and first sacral foramina is identified, which is typically within 1 mm of the superior articulating process of the S1 vertebra. After burring the pilot hole, the S1 pedicle is cannulated with a curved awl with a 30° to 40° medial trajectory aiming for the superior aspect of sacral promontory. The pedicular wall integrity is palpated and confirmed with a ball tip probe in the usual fashion, followed by the placement of the screw. The cannulated pedicle does not need to be tapped unless using larger screws measuring 8 mm or more.

### Pelvic Instrumentation

Pelvic instrumentation includes a MEI screw and a S2AI screw. The decision of which of the two screws is placed first depends on which starting point aligns with the main vertical rod of the construct. Typically, the MEI screw aligns with the main vertical rod and the S2AI screw aligns with the second rod, which would then be placed medial to the first rod. If the S2AI screw is aligned with the lumbar instrumentation, the second rod may run laterally into the MEI screws.

### Medialized Entry Iliac Screw

**Figure 7:**
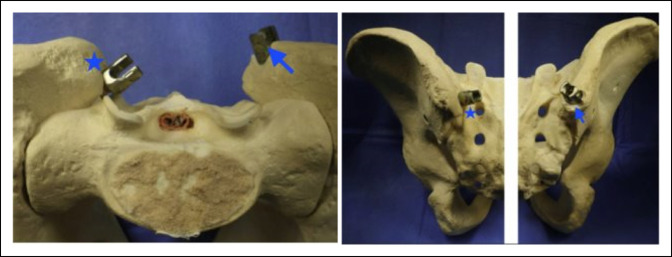
Photographs showing the difference between the conventional prominent iliac bolt (arrow) and the medialized entry screw (star).

For primary pelvic fixation, the conventional iliac screw starts at the posterior superior iliac spine. However, this insertion point is associated with prominence of the instrument, leading to pain and wound issues. (Figure 7). To avoid these issues, the senior author uses a MEI screw. The insertion point is deep to the posterior superior iliac spine on the inner table of the iliac bone just lateral to the sacroiliac joint and midway between the S1 and S2 sacral foramina. The muscular attachments and soft tissues are carefully detached, and this area is exposed using electrocautery. The starting pilot hole is made with a high-speed Burr. The screw trajectory is obtained using a straight Steffee pedicle screw probe; this specific probe is used because of its blunt tip, decreasing the risk of inner/outer table breech during pelvic instrumentation. The trajectory is typically angled about 20 to 45° caudal and 30 to 45° lateral. Gently advancing the probe should allow for the trajectory to stay between the inner and the outer tables of the ilium. The surgeon should be cautious as the natural tendency is to drop the hand to avoid breaching medially into the pelvis, which may inadvertently result in a lateral iliac wall breach. The surgeon's hand should be elevated while advancing the probe and palpating cancellous bone to avoid a lateral breach. The probe is advanced to 70 millimeters. A robust straight ball-tipped feeler probe is then used to advance the trajectory in the cancellous bone. The desired length is usually between 80 and 100 millimeters, and the desired diameter is 9 or 10 millimeters. An 8-millimeter tap is then used to decrease the stresses on the screw during insertion.

### S2 Alar Iliac Screw

For secondary rod fixation, a point between the S1 and S2 dorsal foramina is selected medial to the previously placed primary MEI screw, thereby allowing medial placement of the secondary rod. A standard S2AI screw trajectory is used, traversing the SI joint obliquely aiming toward the greater trochanter with an estimated angulation of 30 to 45 degree of pedicle finder with respect to dorsal sacral surface.^17^ After assessing its integrity with a ball tip probe, the cannulation is then tapped followed by placement of a long iliac screw typically measuring 80 to 100 millimeters with a diameter of 9 millimeters.

After completion of the foundation of the construct bilaterally, bilateral teardrop views are taken with fluoroscopy to check the positioning of the MEI and S2AI screws. The teardrop view is obtained by rolling the C-arm 30 to 40° over the table and then tilting the C-arm 30 to 40° toward the patient's head. The image obtained in this position shows the column of bone extending from the posterior superior iliac spine to the anterior inferior iliac spine, whereby the overlap of the inner and outer cortices of the ilium creates the shape of a teardrop. The contralateral side can then be obtained by rolling the C-arm back to neutral and then 30 to 40° away from the table while maintaining the same 30 to 40° tilt toward the patient's head. The instrumentation is then run cranially using standard landmarks and pedicular trajectories to the upper instrumented vertebrae. A freehand pedicle screw placement technique is used.^18^

After instrumentation is completed, either AP and lateral fluoroscopy or intraoperative CT can be done to assess for screw malposition and ensure appropriate placement of all pedicle screws and pelvis instrumentation after the freehand technique.^19^ In addition, triggered electromyography (EMG) is always done with each pedicle screw to assess for pedicle breach and confirm the safety of the neurologic structures.^20^

This is followed by the preoperatively planned decompression and/or osteotomies with restoration of lordosis. After obtaining the desired lordosis, the rod is then contoured aiming to replicate the desired lordosis followed by dialing in of the appropriate thoracic kyphosis.

Next, the secondary rod is placed by anchoring it into the S2AI screws bilaterally and then connecting with the primary rod using lateral connectors or connecting to medially placed screw in the case of Roy Camille trajectory screws. At least three side-to-side open connectors are generally used on each side to obtain the desired stiffness and stability of the construct. This is followed by final tightening of the set screws starting with the primary construct, followed by the connector-primary rod interface and finally the secondary rod. This sequence is maintained to ensure prevention of undue stress at the connector-primary rod interfaces because they are the weakest point in the construct. The secondary rods should cross to at least two levels above the highest osteotomy site or two levels above the thoracolumbar junction. Decortication is done, and bone graft is added during this procedure.

## Cases

### Case 1

A 57-year-old man with a history of Parkinson disease and prior involvement in a motor vehicle collision presented with progressive back pain, stiffness, and postural decline over several years. Full length, AP, and lateral radiographs are shown at the preoperative, 1 month postoperative, and 20 months postoperative time points (Figure 1). Standing global AP and lateral radiograph using slot-scanning device (EOS) imaging revealed a preoperative thoracolumbar levoscoliosis with a Cobb angle of 43°. An anterior lumbar interbody fusion with hyperlordotic cages was initially planned to achieve anterior column support and sagittal plane correction. The presence of a pelvic kidney and low bifurcation of retroperitoneal vasculature, visualized on axial and sagittal CT, precluded a posterior approach. The patient underwent a T3-pelvis posterior fusion with posterior column osteotomies and a quad-rod technique to increase the stability of the construct (Figure 2). Postoperative radiographs and CT reconstruction demonstrate correction in the coronal and sagittal planes and placement and trajectory of the pelvic instrumentation and iliac screws. The positioning of the anatomic iliac and S2 pelvic screws is shown on fluoroscopic and intraoperative imaging.

### Case 2

A 76-year-old man who underwent gastric bypass for morbid obesity, with postoperative BMI of 31.5, prior L3-L5 laminectomy and posterolateral and interbody fusion, and subsequent revision laminectomy and fusion L2-S1 for footdrop presented with worsening low back pain. A positive sagittal vertical axis of 12.1 cm is shown on sagittal imaging. Pelvic incidence was measured at approximately 70° with a lumbar spine lordosis of approximately 38° (Figure 3). Pseudarthrosis at L2-L3 and peri-instrumentation halos at L2 and L5 were noted on CT (Figure 3). An L5-S1 anterior lumbar interbody fusion with the use of a 30° hyperlordotic cage for restoration of lumbar lordosis and subsequent revision of posterolateral fusion from T10 pelvis (Figure 4) were done. Fluoroscopic teardrop views and intraoperative views demonstrate appropriate placement of the iliac (white star) and S2AI (blue star) screws on each side. Iliac screw trajectory and pelvic instrumentation are shown in the CT and reconstruction. Sagittal balance was restored on the immediate postoperative lateral radiograph and was maintained 6 months postoperatively (Figure 4).

### Case 3

A 71-year-old male with ankylosing spondylitis presented with neck, back, groin, and thigh pain, worsening forward-leaning posture and difficulty with horizontal gaze. Preoperative standing lateral radiographs showed sagittal imbalance with a positive sagittal vertical axis of 11.6 cm (Figure 5). Pelvic incidence was measured at approximately 40° with a lumbar spine kyphosis of approximately 6°. The spine was rigid secondary to ankylosing spondylitis. A pedicle subtraction osteotomy at L4 was done to achieve the lordotic correction. The proposed quad-rod technique was used to secure the achieved correction. Teardrop and lateral intraoperative images demonstrate iliac and S2AI screw placement (Figure 5). Postoperative radiographs demonstrate improved lordosis. 3D reconstruction demonstrates quad-rod instrumentation placement (Figure 6).

## Discussion

Adult spinal deformity treatment is a challenge for spine surgeons. In populations with increasing life expectancy, the incidence of adult spinal deformities may continue to increase. Spine surgery for ASD is a costly intervention. The optimization of techniques and approaches to ensure cost-effective care is essential. Etiology of the deformity can be purely degenerative or can be iatrogenic in cases performed with Harrington rods and similar implants. Short segment decompression and fusion in the lower lumbar spine can contribute to deformity if the surgical planning and correction does not account for sagittal alignment. A degenerative spine, with decreased disk height, which undergoes fusion without proper restoring of the lost disk height(s), can result in flat back syndrome. Correction of the deformity may require a long construct and a lower lumbar spine osteotomy to restore the sagittal alignment. The goal is to achieve fusion of the spine in the corrected position,^21,22^ with the highest stress zone being at the lower lumbar spine and the lumbosacral junction. Even with pelvic fixation using one iliac bolt and one rod on each side, high incidence of nonunion at the L5-S1 level is reported.^23^ This manifests in the form of increased low back pain, implant pull out, or rod breakage. Inserting two traditional iliac bolts is not ideal as it requires two more offset connectors, which can act as a weak link at the caudal aspect of the construct. This is in addition to the pain, soft tissue, and wound issues associated with these screws which may necessitate revision surgery and removal. Long spinal constructs are associated with a high incidence of pseudarthrosis resulting in instrumentation failure at proximal or distal ends.^23,24^ The incidence of pseudarthrosis may correlate with the surgical technique rather than malalignment, the need to determine an optimal surgical approach to ASD spinal surgery.

Instrumentation failure is routinely recognized as a surrogate marker for pseudarthrosis. The technique described in this report is used to augment long spinal constructs especially in elderly patients with high BMI, osteopenia, increased frailty scores, and other comorbidities who require long constructs because of sagittal malalignment. Furthermore, increasing sagittal decompensation in elderly population also requires increasing use of osteotomy techniques, including three-column osteotomy techniques. This results in higher incidence of pseudarthrosis leading to an increased risk of instrumentation failure in this population.

Adult degenerative scoliosis can also be included in the abovementioned cohort with patients frequently requiring long spinal constructs and multiple osteotomies requiring sacroiliac (SI) anchoring. Formerly, SI fixation was obtained by traditional iliac screws and/or S2AI screws. These techniques can be used for good effect by running multiple screw rod constructs up along the length of construct. This requires careful planning preoperatively by using the available space for placement of a two large diameter screw heads on each side. The intended advantage of such instrumentation architecture is two-fold, first and foremost by increasing rigidity across the lumbosacral transition, suggested by Kelly et al^25^ in in vitro testing, thus promoting fusion across the lumbosacral junction and second to decrease rod fracture and thus potentially obviating the need for revision surgery, as demonstrated by Shen et al^26^ in a cohort of 32 patients with 8.3% rate of rod breakage, however, with none of the patients requiring revision surgery.

Construct augmentation with multiple rods has been increasingly used and described over the past one to 2 decades with varying architecture.^27,28^ A cadaveric study demonstrated greater stability when using a 4-rod technique compared with S1 or S1 plus iliac screws.^29^ A comparative study by Hyun et al^30^ showed superiority for multirod constructs with a three-column osteotomy compared with a dual-column approach.

In a similar study, Yamato et al^31^ also highlighted the reduction of rod fracture in multirod constructs in comparison with a dual-rod approach. Patients in the three-column approach showed notable reductions in rates of rod breakage and revision surgery for pseudarthroses versus two-column osteotomies. Most of these descriptions include secondary systems comprising rods and connectors which differ from our technique. Our described architecture involves individual SI anchoring, in the form of separate dedicated S2AI screw, thus promoting increasing rigidity across the construct foundation via stress distribution over two iliac bolts. This increased rigidity may have implications on fusion rates because a stiffer construct may decrease motion at the lumbosacral junction and lower the risk of mechanical failure, thereby promoting protective factors for fusion.^25^ Kelly at al. found increased stability and flexibility with the four-rod model compared with the conventional two-rod construct.^32^ A recent retrospective study by Villazón et al compared a pelvic fixation with a multirod construct to two-rod constructs in the setting of ASD and found no difference in complication rates in the use of additional rods.^33^ This study of 33 patients with multirod fixation and 33 patients with a dual-rod approach found a higher rate of rod breakage and revision surgery in the dual-rod cohort. A retrospective study by Merrill et al^34^ compared 15 patients in the dual-rod group and 16 in the multirod group and found that the dual-rod construct cohort exhibited a higher rate of pseudarthroses. Despite a limited sample size, the authors concluded that increasing construct rigidity with multiple rods has been associated with superior fusion outcomes.

This technique has been previously described by the senior author.^10^ Therefore, this study aims to describe the technical nuances of the technique with recent clinical examples. This technique can also be used in short lumbosacral constructs if mechanical demands dictate the need for a more robust construct. The potential clinical non-ASD scenarios would be cases requiring extensive sacral osteotomy in neoplasm resection. Other cases would include high-grade spondylolistheses requiring surgical management and/or revision cases with lumbosacral junctional pseudo arthrosis, as well as traumatic case of unstable U/H-shaped sacral fractures with spinopelvic dissociation.

The safety and complication profile, although outside the purview of this study, is comparable with the expected norm for such procedures. The abovementioned architecture relies on established instrumentation techniques. Additional work on the long-term clinical outcomes of the technique is warranted. Future work validating the rigidity of this construct could include biomechanical studies comparing the quad-rod technique with more traditional dual-rod constructs using either traditional iliac bolts or S2AI fixation alone. Furthermore, long-term follow-up of patients with ASD treated with the quad-rod construct will provide additional insight into rates of nonunion and/or instrumentation failure with this technique. Although the increased stiffness of the quad-rod construct may decrease rates of mechanical failure, the potential association between construct stiffness and bony fusion requires additional investigation. The quad-rod construct may improve fixation by providing alternative traversing of rods through the lumbosacral junction. The quad-rod construct described in this study specifically proposes a method involving four rods, each of which is independently anchored to its own dedicated pelvic bolt. Other multirod constructs use side-to-side connectors to attach a supplementary rod along the side of one or both main rods. The construct is anchored to the pelvis using one or two pelvic screws on each side. These other constructs rely on the two main rods, and their anchor points as the foundation of the construct. The supplementary rod is dependent on the cross connectors used near the pelvic bolts. Our method increases redundancy in pelvic fixation and relies less on the integrity of the two main rods. In addition, the supplementary rods are not functionally dependent on a single pair of cross connectors at the base of the construct. These three cases and a review of the literature support the implementation of the quad-rod augmentation technique as a potential surgical technique for thoracolumbar spinal constructs in patients with complex ASD.

## Conclusion

Quad-rod augmentation of long thoracolumbar spinal constructs with two independent SI anchoring points is potentially an effective technique, with the objective of increasing lumbar sacral construct rigidity, which may promote fusion rates and thereby decreasing revision. The described technique may serve as an effective tool to treat patients with ASD.
